# Research and application of single-cell sequencing in tumor heterogeneity and drug resistance of circulating tumor cells

**DOI:** 10.1186/s40364-020-00240-1

**Published:** 2020-11-10

**Authors:** Zhe Dai, Xu-yu Gu, Shou-yan Xiang, Dan-dan Gong, Chang-feng Man, Yu Fan

**Affiliations:** Cancer Institution, Affiliated People’s Hospital of Jiangsu University, No.8 Dianli Road, Zhenjiang, Jiangsu Province 212002 People’s Republic of China

**Keywords:** Tumor heterogeneity, Circulating tumor cells, Drug resistance, Single-cell sequencing

## Abstract

Malignant tumor is a largely harmful disease worldwide. The cure rate of malignant tumors increases with the continuous discovery of anti-tumor drugs and the optimisation of chemotherapy options. However, drug resistance of tumor cells remains a massive obstacle in the treatment of anti-tumor drugs. The heterogeneity of malignant tumors makes studying it further difficult for us. In recent years, using single-cell sequencing technology to study and analyse circulating tumor cells can avoid the interference of tumor heterogeneity and provide a new perspective for us to understand tumor drug resistance.

## Background

Malignant tumor is a common disease whose incidence increases yearly. It has become a considerable threat to human health. In recent years, the five-year survival rate of patients has substantially improved with the widespread use of anti-tumor drugs. However, the vast majority of tumors have drug resistance, which is a large barrier to treatment [1]. Heterogeneity of tumor cells is considered an important cause of drug resistance. In the past, we studied drug resistance in tumor cells through high-throughput sequencing based on numerous mixed cell samples, which ignored the heterogeneity of tumor cells and resulted in the dilution of the genetic characteristics of low-abundance but functionally essential cells such as circulating tumor cells (CTC). In recent years, the research methods for CTC have become more diversified. Therefore, single-cell sequencing of CTC can analyse the information of a single cell genome, transcriptome and epigenetic group, which reduces the interference of tumor heterogeneity [2] and provides a new perspective for understanding the drug resistance of tumors.

## Relationship between tumor cell heterogeneity and drug resistance

**Fig. 1:**
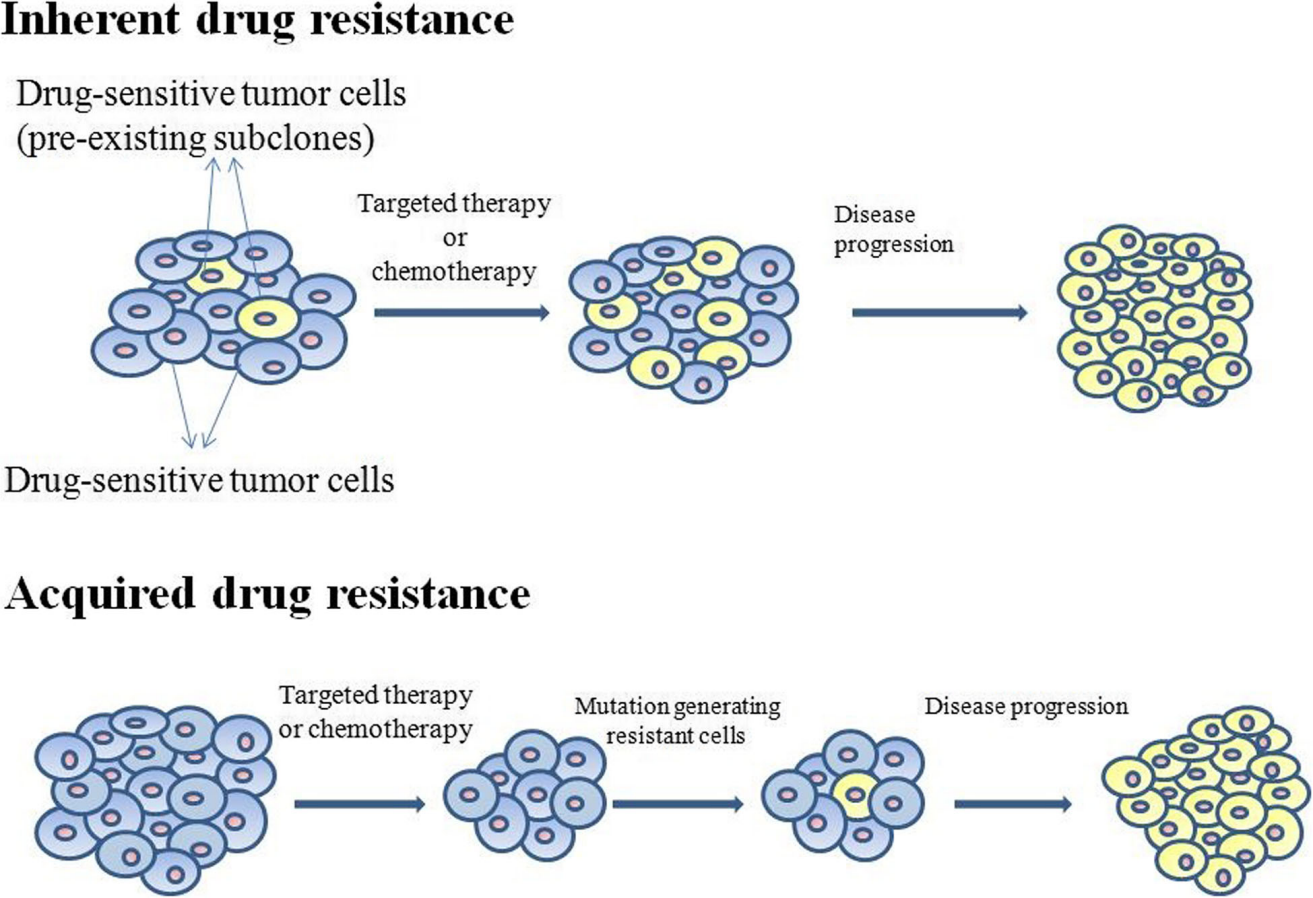
Two mechanisms lead to drug resistance in tumors: inherent drug resistance and acquired drug resistance

**Fig. 2:**
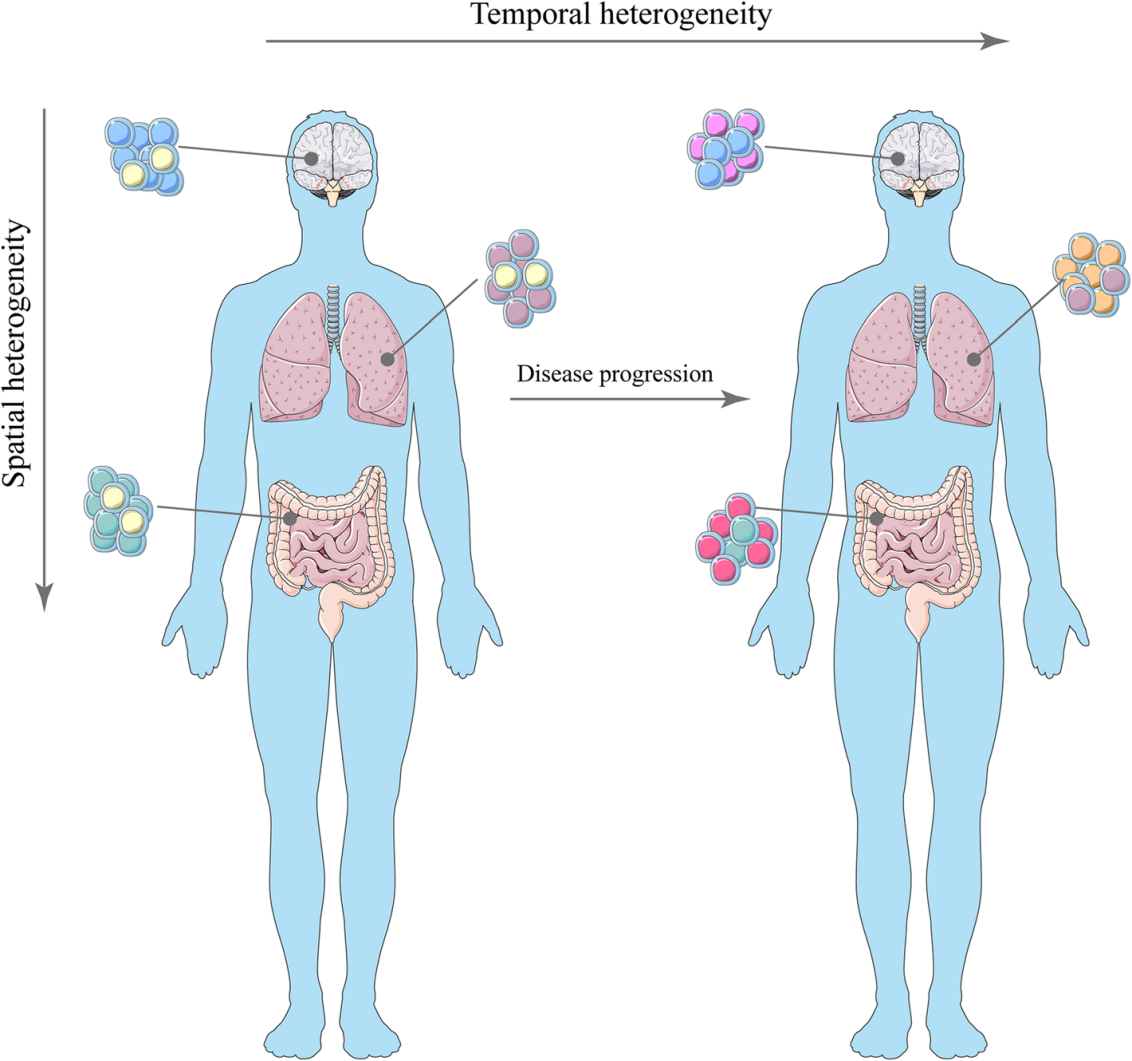
Spatial heterogeneity and Temporal heterogeneity

Scientists currently believe that two mechanisms lead to drug resistance in tumors: inherent drug resistance and acquired drug resistance (Fig. 1). Inherent drug resistance is the existing drug resistance before the use of anti-tumor drugs. Acquired drug resistance occurs during or after treatment. Inherent drug resistance may arise from rare pre-existing subclones, whereas acquired drug resistance is an acquired new mutation [3]. After multiple divisions and proliferation of tumor cells, their progeny cells show inconsistencies in genomic and biological characteristics, and this inconsistency makes several biological characteristics of tumor cells different, which is called tumor heterogeneity. Tumor heterogeneity can be divided into inter-tumor heterogeneity and intra-tumor heterogeneity. The current research focus is on heterogeneity within the tumor. Heterogeneity in the tumor includes spatial heterogeneity and temporal heterogeneity (Fig. 2). In tumors, different cellular clones at different spatial sites lead to spatial heterogeneity. Tumor cells change with time, which is the temporal heterogeneity of tumor cells [4]. tumor cells also affect the stroma, immune cells and other cells, which constitutes the heterogeneity of the tumor microenvironment (TME) [5]. Numerous studies have shown that tumor heterogeneity is an important cause of drug resistance in tumor cells [6]. For example, cells with strong drug resistance will gradually replace cells sensitive to drugs with the progress of chemotherapy [7]. Thus, we need to have a deeper understanding of tumor heterogeneity.

## Value of single-cell sequencing in the study of tumor cell heterogeneity

Important information such as mutation status, epigenetic status and related protein expression levels of tumor cells may be expressed only in few cells or even in a single cell [8]. Heterogeneity is ignored if mixed tumor cells are used for analysis. Studying the drug resistance of tumor cells at the single-cell level is important. Single-cell sequencing technology refers to a technique for sequencing the genome and transcriptome at the single-cell level. Compared with previous sequencing methods, it can perform a more thorough analysis of healthy cells and tumor cells [9]. It can also identify previously unknown cell types [10, 11]. Thus, it can better reveal the heterogeneity of tumor cells at the cellular and molecular levels. Using single-cell sequencing technology to study the heterogeneity of tumor cells has been widely practiced in malignant tumors such as breast cancer, melanoma and lung cancer [12–16].

## Single-cell sequencing of CTC and drug resistance

As mentioned earlier, the heterogeneity of tumor cells, especially the transcriptome information, including time and space limitation, is likely to change constantly. Hence, studying the heterogeneity of tumor cells dynamically can better explore the problem of tumor drug resistance [17]. The main approaches of obtaining tissue specimens are tissue biopsy and cell puncture [18], which is an invasive procedure with the risk of tumor spread, especially in patients with advanced cancer and multiple metastases [19]. Moreover, researchers may not be able to acquire sufficient experimental standard quality tissue specimens for various reasons. CTC, a type of tumor cell that is separated from the primary focus or metastasis of solid tumors and enters the peripheral blood circulation, has gradually come into people’s field of vision. Studies have confirmed that CTC has characteristics similar to those of tissue cells at the single-cell sequencing level [20]. Extracting tumor cells from peripheral blood has the advantage of being less invasive. In the state of peripheral blood circulation, various types of tumors are relatively uniform, which can more accurately reflect the temporal and spatial heterogeneity of tumor cells [21]. Therefore, increasing studies are conducted on tumor resistance through CTC. Scholars study choriocarcinoma [22], colorectal cancer [23–26], liver cancer [27], lung cancer [28, 29], breast cancer [30, 31], lymphoma [32], gastric cancer [33] and other malignant tumors through CTC to explore the drug resistance of tumor cells. However, most current CTC analyses are based on CTC epithelial biomarkers, and epithelial biomarkers may not be expressed in several tumor types [34]. Analyses have not been studied at the genomic level, thus lacking individual CTC characteristics or genetic characteristics of patients [35]. CTC accounts for a small proportion of blood, and only a few dozen CTCs can be obtained at a single time. The genetic material isolated from a single cell is minimal. Applying traditional sequencing methods to this kind of research is challenging. The minimal raw materials of cells hinders the analysis of tumor cells. Although the whole genome amplification (WGA) technology has been developed for decades, the previous WGA technology has a greater difficulty in achieving high-quality genome amplification [36]. The application of single-cell sequencing to analyse CTC can study the information at the gene level in more detail to explore the relevant mechanisms of drug resistance in tumor cells further, accurately predict the effect of anti-tumor drugs and formulate more effective drug regimens [37].

## Single-cell sequencing of CTC to explore the mechanism of drug resistance in tumor cells

In recent years, scientists studied CTC through single-cell sequencing technology and discovered new mechanisms of drug resistance of tumor cells. Miyamoto et al. [38] isolated 133 CTC from blood samples of patients with prostate cancer and performed single-cell RNA sequencing on CTC, original tumor samples and samples of prostate cancer cell lines. Previous studies showed that the androgen receptor (AR) pathway is a first-line therapeutic target for prostate cancer. However, Miyamoto found that AR point mutations associated with altered signalling are not common in patients with castration-resistant prostate cancer (CRPC). tumor heterogeneity exists between patients such that different CTCs have different or more mRNA splicing changes. Compared with metastatic prostate cancer without enzalutamide (an AR inhibitor), non-classical Wnt signalling is considerably enriched in prostate CTC treated with enzalutamide during the progression of radiological imaging or prostate-specific antigen. CTCs with low glucocorticoid receptor (GR) expression levels are also enriched in non-classical Wnt signals in patients with enzalutamide progression but not in CTCs with high GR levels. Studies have shown that non-classical Wnt signalling may be a mechanism of CRPC resistance.

Anaplastic lymphoma kinase (ALK) gene, first discovered in a subtype of anaplastic large cell lymphoma, is responsible for encoding a receptor tyrosine kinase (RTK) called ALK. The common pathogenic mutation in the ALK gene is gene rearrangement, which can lead to tumors. ALK gene fusion often occurs in a subset of patients with non-small-cell lung cancer (NSCLC); thus, ALK inhibitors are useful in the treatment of such patients. However, such patients will inevitably become resistant to ALK inhibitors, leading to treatment failure. Pailler et al. [39] studied drug resistance mutations in CTCs isolated from 17 patients with ALK-rearranged NSCLC (14 were resistant to crizotinib, and 3 were resistant to loratinib) and studied more than 48 cancer-related genes and 14 ALK mutation regions at the single-cell level. Genomic heterogeneity was found in CTC from patients with crizotinib-resistant ALK rearrangement. In the ALK-independent pathway, the kirsten rat sarcoma viral oncogene and TP53 pathways play a significant role. In a patient resistant to loratinib, Pailler identified two ALK multiple mutations, which are a ‘targeted’ resistance mechanisms. These mutations are likely the mechanism of resistance of patients with ALK-rearrangement and NSCLC to ALK inhibitors.

According to Miyamoto’s sequencing results, Schissler [40] studied the mechanism of prostate cancer resistance from another perspective and argued that Miyamoto’s single-cell sequencing results lack a functional explanation, and the approach to sequencing does not fully apply to small populations of cells such as CTC. Schissler et al. designed an experimental model to simulate transcriptome dynamics by analysing the cell–cell statistical distance of aggregation within a biomolecular pathway to identify differentially expressed pathways associated with drug resistance. They designed a new aggregation method, cell-centric statistics method, and proved its effectiveness in predicting a single CTC’s drug resistance. They found that five types of pathways are substantially overexpressed in the CTC of patients with enzalutamide resistance. Syndecan-4-mediated signal transduction pathways play an important role in the resistance mechanism.

Most breast cancers express estrogen receptor (ER); thus, targeted therapy has become the first choice for the treatment of ER-positive breast cancer [41]. Selective ER modulators or decomposers can be used to target the ER pathway, and aromatase inhibitors (AI) and other drugs that cause estrogen deficiency can be used for treatment. Studies have shown that estrogen receptor 1 (ESR1) mutations are known resistance mechanisms of tumor cells to AI or gonadotropin-releasing hormone analogues. Franken et al. [42] performed CTC single-cell sequencing on 46 patients with metastatic breast cancer and found that mutations in ESR1 (including known hot spot mutations and new mutations) are only present in patients receiving estrogen deprivation therapy (EDT). By contrast, no mutations were detected in patients not receiving EDT or other types of therapy. The researchers believe that the newly discovered mutations could lead to resistance to targeted drugs because the newly discovered mutations are found only in patients receiving targeted therapy and affect highly conserved amino acids. Hong et al. [43] believed in an interaction between genetic and non-genetic factors in the development of resistance to endocrine therapy in breast cancer. They used single-cell RNA sequencing to analyse tumor cells and defined a subpopulation of cells that may be pre-resistant to endocrine therapy. This subpopulation is substantially expressed in CTC. Transcription reprogramming and copy number changes to achieve complete drug resistance also reveal a multi-step drug resistance mechanism that interacts with genetic and non-genetic factors.

Researchers [44] found that the emergence of KRAS mutant clones is the second mechanism of colon cancer resistance through the single-cell analysis of CTC in patients with colon cancer. Researchers [45] found another acquired epidermal growth factor receptor (EGFR) extracellular region mutation (S492R) in the CTC of patients with colorectal cancer, which can prevent cetuximab (an EGFR blocking antibody), enabling its resistance to cetuximab.

Many studies fully demonstrated that using single-cell sequencing technology to analyse CTC can better study the mechanism of tumor drug resistance.

## Single-cell sequencing of CTC to predict drug resistance in tumor cells

Scientists currently believe that drug resistance of tumor cells is a complex biological behaviour with continuous evolution and dynamic change in tumor development. In this process, multiple factors affect the drug resistance of tumor cells with dynamic changes over time. The use of single-cell sequencing to monitor the abnormal signal pathway of CTC can predict drug resistance and realise individualisation and precise treatment.

Malignant tumor cells involve a series of genomic changes, including copy number variations (CNVs), single nucleotide variations (SNVs) and insertions/deletions (indels). In lung cancer, the timely detection of mutations that lead to drug resistance is essential for selecting the appropriate treatment for patients. Lung cancer with EGFR mutation is initially well treated with tyrosine kinase inhibitor [46], but drug resistance is inevitable with drugs such as Gefitinib and Erlotinib. This resistance can be overcome in combination with other drugs [47]. Therefore, the EGFR expression profile of patients with NSCLC must be monitored in time to capture the dynamic time changes of the disease for treatment. Ni et al. [48] effectively solved the problem of insufficient DNA content in CTC by using multiple annealing circular loop amplification (MALBAC) and achieved genome-wide amplification. Thus, CNV and SNV can be accurately measured in a single cell. Mutations in the EGFR gene were found in the CTC of one patient, and PIK3CA mutations were detected in the CTC of this patient through single-cell genome sequencing of 11 patients with NSCLC. Studies have shown that PIK3CA mutation is associated with erlotinib resistance [49]. The patient developed rapidly after one month of treatment with erlotinib. Ni believed that the single-cell sequencing of CTC could effectively predict the drug resistance of anti-tumor drugs and facilitate the further guidance of drug use. Tan et al. [50] also performed EGFR mutation detection on a single CTC in seven patients with NSCLC, such as L858R and T790M. Compared with the matched tumor biopsy results, the results showed the right consistency between the two. Thus, single-cell analysis can provide a more accurate disease map, which is crucial for the timely detection of mutations leading to drug resistance and the selection of appropriate treatment options for patients. Similar conclusion can also be made in single-cell sequencing of CTC in prostate cancer. A single-cell research of CTCs from blood samples of patients with localised high-risk prostate cancer showed 202,241 SNVs and 137,407 indels. The researcher explored the effect of the SNVs identified in CTCs on drug response targets and found that nine genetic variations are associated with the response to docetaxel, and 48 SNVs influence drug response for 24 known cancer drugs. A gene set enrichment analysis was carried out to study the correlation between CTC-shared copy number alterations (CNAs) and pathways. The results showed some gene amplification affecting DNA damage repair pathways, which could result in chemotherapy resistance [51].

For small-cell lung cancer, no convincing treatment plan other than chemotherapy currently exists. However, its drug resistance also leads to the failure of many treatments, and several patients even develop drug resistance after the first use of chemotherapy drugs. Clinically, patients with small-cell lung cancer can be divided into a chemotherapy-resistant group and a chemotherapy-sensitive group according to the response within 3 months after initial chemotherapy. Tumor cell adhesion is poor in small-cell lung cancer. The expression characteristics of CTC may be higher than those of other cancer types. Su et al. [52] performed whole genome sequencing detection of CTC in 10 patients with small-cell lung cancer who received standard chemotherapy regimens of etoposide + platinum (cisplatin, neda platinum or carboplatin). Amongst the 91 CTCs selected, the CNA spectra of patients’ CTC were found highly consistent, which also confirmed that CTC could be used as an ideal sample for clinical correlation analysis of patients with cancer. Based on the sequencing of a single CTC, the researchers established a CNA score to analyse the correlation between the CNA data and the efficacy and survival of first-line chemotherapy. Su et al. used the CNA score as a standard to predict the clinical subtypes of patients, correctly predicting 20 out of 25 patients with chemotherapy resistance and 15 out of 16 patients with chemotherapy sensitivity. Similarly, Carter et al. [53] detected CNA on 88 CTC cells isolated from 13 patients with small-cell lung cancer and generated a CNA-based classifier, which was then tested on another 18 patients. After verifying 112 CTC cells from 18 patients, the accuracy was found to be 83.3%. Studies have sufficiently demonstrated that the CTC single-cell sequencing technology can be used to determine whether patients are sensitive or resistant to chemotherapy drugs, providing theoretical support for the realisation of accurate treatment.

Single-cell genome sequencing can provide genetic heterogeneity and cell lineage information, whereas single-cell transcriptome sequencing can more dynamically represent the sum of all RNAs produced by a species or a specific cell under a functional state, which can better define the current table of cells type. Cheng et al. [54] used hydro-seq technology to conduct transcriptome sequencing of CTC and selected 666 CTCs from 21 patients with breast cancer. The drug targets of breast cancer, including ER, AR and human epidermal growth factor receptor 2 were successfully detected from CTC, and the full transcriptome analysis of CTCs was effectively realised to study the heterogeneity of tumor cells further. This finding also proves that CTC single-cell transcriptome sequencing is an effective method to analyse the molecular characteristics of tumor cells and can be used for treatment selection and patient monitoring.

## Single-cell sequencing of CTC to investigate the association with drug resistance and TME

As mentioned previously, tumor heterogeneity is an important theoretical basis for drug resistance. Tumor heterogeneity is not only affected by tumor cells themselves but also closely related to infiltrating immune cells [55], endothelial cells forming blood vessels [56], cancer-associated fibroblast (CAF) [57] and extracellular matrix [58]. These internal and external environments that are closely related to the development of tumor and tumor cells together constitute the TME. Therefore, the study of TME has become an important means to explore the drug resistance of tumors. Considerable research supported the viewpoint that TME could influence the therapy response by different mechanisms. A recent study reported that TME fibroblasts can be differentiated into CAFs by tumor-derived exosomes, which promote drug-resistant phenotypes [59]. Extracellular vesicle-mediated intercellular communication has also been proven to play a crucial role in the response to anti-tumor treatments [60]. In addition, tumor-associated macrophages and other stromal cells from TME could affect tumor resistance [61]. In recent years, studies showed that the detection of CTC could reflect its relationship with TME and the heterogeneity of TME [62, 63]. This technique applied in CTC showed the potential to explore the association with drug resistance and TME with the development of single-cell sequencing. Heather et al. [64] applied single cell RNA sequencing to identify two populations of CTCs amongst 1707 cells from 14 patients with breast cancer. According to transcriptome analysis, CTCs could be divided into two subgroups: one is rich in transcripts that are characterised by estrogen responsiveness and increased proliferation, and the other is rich in transcripts characterised by reduced proliferation and epithelial–mesenchymal transition (EMT). A cell–cell communication tool was used to investigate the association between CTCs and TME. The results showed that CTCs with increased EMT markers have a more diverse pattern of interaction. The gene expression pathway activation analysis showed that a CTC population with EMT characteristics has a lower likelihood of activating the apoptotic pathway, which could lead to drug resistance. Heather’s study indicates that the single-cell sequencing of CTC can be used to further explore the tumor microenvironment and tumor drug resistance.

## Conclusion

The single-cell sequencing of CTC is a burgeoning technology, but it is not yet fully mature and has several flaws. For example, fewer nucleotide feedstocks may be more likely to cause biological noise than conventional sequencing. The latest linear amplification via transposon insertion method [65] can address this problem. Another limitation is that very few materials are available. For example, in single-cell transcriptome sequencing of CTC, each cell contains an average of only about 10 pique total RNA, in which the mRNA is only about 0.1 pique; people think that CTC is not found in the blood of all patients with cancer, especially patients with early cancer [66]; hence, the single-cell sequencing of CTC is still not used on a large scale. Moreover, whether CTC can fully express all tumor information remains controversial, and studies found no apparent relationship between tumor tissue and CTC [67]. To what extent CTC represents the corresponding tumor remains controversial, such that people [68] proposed combining single-cell sequencing with traditional sequencing technology and further analysing the results to reduce the error. The single-cell sequencing of CTC will inevitably lead to a partial loss of spatial information of tumor cells. The spatial location of interactions between various tumor cells and cells in the TME is also critical to the development of tumors. Single-cell sequencing is necessary for our future research on tumor resistance to avoid the spatial information loss caused by CTC.

In addition, the bioinformatics and computational methods of single-cell sequencing remain unable to match the corresponding data fully, and the new single-cell sequencing data make the existing analysis tools appear impractical. At present, single-cell sequencing technology has also produced unprecedented data types. For example, two new sequencing algorithms [69, 70], which consider the spatial analysis of variance of malignant tumors, have been proposed recently. Therefore, the expanding data are also a considerable challenge for single-cell analysis.

Although the single-cell sequencing of CTC remains immature, this cross-era technology has its natural advantages [71], and we believe that several problems will be gradually solved with the continuous optimisation of genome-wide, transcriptome amplification methods and the rapid development of bioinformatics.

The single-cell sequencing of CTC contributes to the study of genetic heterogeneity and drug resistance of tumor cells. The single-cell sequencing of CTC certainly provides more powerful tools to understand many of the remaining mysteries of cancer cell resistance.

## Data Availability

Not applicable.

## Abbreviations

AI: Aromatase inhibitors
ALK: Anaplastic lymphoma kinase
ALCL: Anaplastic large cell lymphoma
ANOVA: Analysis of variance
AR: Androgen receptor
CAF: Cancer-associated fibroblast
CCS: Cell-centric statistics
CRPC: Castration-resistant prostate cancer
CTC: Circulating tumor cell
CNA: Copy number alteration
CNV: Copy number variations
EDT: Estrogen Deprivation Therapy
EGFR: Epidermal growth factor receptor
EMT: Epithelial–mesenchymal transition
ER: Estrogen receptor
ESR1: Estrogen receptor 1
GR: Glucocorticoid receptor
GnRH-A: Gonadotropin-releasing hormone analogs
HER2: Human epidermal growth factor receptor 2
KRAS: Kirsten rat sarcoma viral oncogene
LIANTI: Linear amplification via transposon insertion
MALBAC: Multiple annealing circular loop amplification
NSCLC: Non-small cell lung cancer
PSA: Prostate specific antigen
RTK: Receptor tyrosine kinase
SNV: Single nucleotide variations
TME: Tumor microenvironment
TKI: Tyrosine kinase inhibitor
WGS: Whole genome sequencing
WGA: Whole genome amplification
